# The political economy of tobacco of Zimbabwe: An analysis of stakeholder perspectives

**DOI:** 10.1371/journal.pgph.0004805

**Published:** 2025-06-30

**Authors:** Shashika Bandara, Artwell Kadungure, Nhamo Nhamo, Tendai Nyashanu, Ashley Chamunorwa, Jeffrey Drope, Matthew Hunt, Alayne Adams, Raphael Lencucha

**Affiliations:** 1 Faculty of Medicine and Health Sciences, Department of Family Medicine, McGill University, Montreal, Canada; 2 Training and Research Support Center, Harare, Zimbabwe; 3 Marondera University, Marondera, Zimbabwe; 4 Independent Researcher, Harare, Zimbabwe; 5 Bloomberg School of Public Health, Johns Hopkins University, Baltimore, Maryland, United States of America; 6 School of Physical and Occupational Therapy, McGill University, Montreal, Canada; PLOS: Public Library of Science, UNITED STATES OF AMERICA

## Abstract

Tobacco continues to be viewed as a path to economic development by some governments, including Zimbabwe, despite widespread knowledge of tobacco’s negative consequences for users, farmers and the environment. Zimbabwe is one of the top five tobacco producers in the world and the largest tobacco producer in the African region. Zimbabwe’s focus on tobacco production creates difficulties for the implementation of the World Health Organization Framework Convention on Tobacco Control (WHO FCTC) which it signed in 2014. Our objective was to understand the political economy of tobacco in Zimbabwe, which can inform the implementation of WHO FCTC. We conducted 23 interviews with government, non-governmental, para-statal and other stakeholders in Zimbabwe’s tobacco sector. The findings illustrate intersecting issues that make tobacco a complex policy issue in the country. Interviewees indicated that WHO FCTC implementation faces barriers due to tobacco being an important economic commodity. Specifically, interviewees highlighted the prioritization of tobacco in government strategic plans and as a priority agricultural crop. Additionally, lack of alternatives and debt obligations makes it challenging for smallholder farmers to shift away from tobacco. However, findings indicate that tobacco production is facing rising challenges including tobacco use among youth, deforestation and farmer health, poverty and debt making the supply and demand measures of the WHO FCTC even more crucial. These challenges may be starting points to engage with the government in order to encourage strategies to move away from tobacco as a key economic commodity.

## Introduction

Globally, every year there are over 8 million deaths associated with tobacco use and second-hand smoke is estimated to cause 1.3 million deaths among non-smokers [1]. Tobacco farming is also harmful to human and planetary health. It contributes to food insecurity occupying 3.2 million hectares of fertile land globally that could be used to grow food crops, while 349 million people face acute food insecurity [2]. Tobacco farming practices pose health risks to growers and those handling the leaf [2]. Tobacco farming also has implications for the climate crisis especially due to deforestation [2]. Despite the harmful impacts of tobacco growing and use, tobacco continues to be viewed as a path to economic development by some governments [3,4]. Yet the promise of economic prosperity associated with tobacco farming has not materialized for smallholder farmers, often leading rural households into perpetual debt [5], and in some instances necessitating government support in the form of poverty alleviation programs [6].

The World Health Organization Framework Convention on Tobacco Control (WHO FCTC) came into force in 2005, focused on reducing tobacco consumption and tobacco supply [7]. Though it acceded to WHO FCTC in 2014, Zimbabwe continues to prioritize tobacco production, especially as a foreign income earner [8–10]. Zimbabwe is one of the top five tobacco producers in the world and the largest tobacco producer in the African region [8,11–13].

In the field of tobacco control, researchers have argued the importance of understanding the political economy to strengthen effective implementation of the WHO FCTC [14,15]. Political economy analysis mainly focuses on the interplay between power, politics, wealth, economics including market behavior, and public policy [16]. Public health policy formulation and implementation are impacted by the political economy of a country [14]. Political economy analysis seeks to identify historical and contemporary contextual factors and how these factors impact power, revenue generation, and policy making and implementation at the national level [14,15]. For example, as will be explained below, historical events and processes, such as colonization, and structures that were built through colonization have trajectories that shape contemporary institutions, policies, and priorities [17]. This paper draws from historical and contemporary contextual factors and key informant interviews to analyze the political economy of tobacco in Zimbabwe.

Considering the contemporary context, in 2023, Zimbabwe exported 233,896 metric tons of tobacco leaf [18], increasing from 220,000 metric tons in 2021 [12]. The government continues to prioritize tobacco within its development plans, setting an agricultural output target of 300,000 metric tons by 2024–25 in its National Development Strategy 1 (NDS1) 2021–25 [19]. Buyers of Zimbabwe tobacco leaf include companies such as British American Tobacco via Northern Tobacco leaf buying company and Tian Ze Tobacco a subsidiary of China Tobacco [20,21]. In 2020, Zimbabwe’s tobacco exports amounted to 16.9% of the total export commodities and 2.4% of the total Gross Domestic Production (GDP) [8]. According to the NDS1, tobacco and gold rank as the main export products of Zimbabwe [19,22]. A Tobacco Value Chain Transformation Strategy approved in 2021 seeks to focus on increased tobacco production, localization of the financing of the crop, production of cigarettes and their exports to increase the value of Zimbabwean tobacco industry to US$5 billion by 2025 [23]. Figures from 2018 suggest that 50,000 small scale growers (6 ha per individual), 8000 small scale commercial growers (148 ha), 70,000 communal growers (12 ha), and 9000 medium- to large-scale growers (318 – 2,200 ha) are involved in tobacco production in Zimbabwe [24]. Importantly, this prioritization of tobacco as an economic commodity runs contrary to Article 17 of the WHO FCTC which requires Parties to reduce supply and support alternative livelihoods for those in tobacco industry [25].

The historical context of Zimbabwe, in particular its almost 100 years of colonization prior to independence in 1980, is important to understand the political economy of commercial tobacco in the country. The British South Africa Company settlers established large farms to grow tobacco for export in what was then called Southern Rhodesia. Despite disruptions caused by World War I, tobacco production grew dramatically between the early and mid-1900s propelled in part by the British Empire’s desire to source tobacco leaf from its colonies rather than from America [26]. The farming of Virginia leaf, the main tobacco export of Zimbabwe, began in 1910 by the colonial settlers who carried significant political power and owned some of the best farming land [13,26]. Colonial settlers reinforced their exclusive rights to tobacco growing through laws such as the Tobacco Licensing Act (1933) that restricted tobacco farming to European owed land [27].

After independence, government supported programs were established to diversify national economic activities. These programs aimed to meet the need for foreign currency and ensure economic stability [22]. The post-independence tobacco trajectory can be broken down into two critical periods: the initial period between 1980–2000 and the post-2000 periods [22]. The dividing line between the two time periods is the landmark Fast Track Land Reform Program (FLTRP) initiated in the year 2000. During the early years of independence (1980–1999), tobacco production was mainly conducted on large commercial farms mostly owned by white farmers. The main actors during this period were Zimbabwe Tobacco Association, Tobacco Industrial Marketing Board (TIMB), Tobacco Trade Association, the Tobacco Research Board, the Agricultural Finance Corporation and the Department of Agriculture Technical Extension Services [22]. While post-independence priorities focused on land redistribution, these efforts stalled due to the need for foreign currency and economic stability.

Largely due to fast-track land re-distribution and related breakdown of the rule of law between 2000–2002, Zimbabwe faced sanctions introduced by the European Union and the United States [28–30]. Affected by sanctions and economic crisis, Zimbabwe saw record inflation rates and low revenues [28,30]. Between 1999–2008, Zimbabwe experienced a 50% decrease in GDP [5,31]. Zimbabwe continues to face sanctions only from the European Union, United Kingdom and the United States, citing Zimbabwe’s human rights record including violent crackdowns against government opposition [32]. There are no UN sanctions on Zimbabwe [33]. African country leaders and other governments such as China, from the beginning, refused to endorse the sanctions against Zimbabwe which has played a role in establishing China and other countries as major trading partners [28]. In August of 2023 the *United Nations special rapporteur on the negative impact of unilateral and coercive measures on the enjoyment of human rights*, called for lifting of sanctions highlighting the adverse impact they have on living conditions and human rights of Zimbabweans at a time of crisis [32]. In March 2024, the US revoked a sanction program administered by its Office of Foreign Assets Control (OFAC) removing restrictions on some state-owned companies and individuals but also saw 11 individuals and three entities, including the country’s sitting president, being sanctioned under the Global Magnitsky Human Rights Accountability Act [34].

Zimbabwe’s *Vision 2030*, unveiled in 2018, highlights the government’s goal to make Zimbabwe an upper middle-income country by 2030 [35,36]. One of the main priorities is to regain investor confidence via policies that focus on “upholding democratic principles, rule of law and property rights,”[36]. Vision 2030 also highlights Zimbabwe’s intention to prioritize private sector led growth which currently includes the tobacco industry [36]. At present, Zimbabwe continues to face severe economic hardship due to high levels of inflation and a volatile currency exchange rate, impacted by many interconnected factors including economic policy, sanctions, and lack of foreign exchange earnings [37]. The projected slowing of real GDP growth (which remained high at 6.5% in 2022) affected by high inflation (at 47.6% in February 2024) and exchange rates also contribute to continuing the economic crisis in Zimbabwe [31,37]. The exchange rate moved from Zimbabwean Dollar (ZWL) 142 on 4 April 2022 to ZWL 26,412 on 4 April 2024 per US dollar [38]. Between January and March 2024, local currency has depreciated more than 70% against the US dollar [31]. As a result, communities in Zimbabwe face extreme poverty [39]. Poverty is high especially in the context of agricultural production affected by persistent inflation, high dependence on low productivity agriculture, impacts of natural disasters, including drought, and the COVID-19 pandemic [35,37,39,40]. Given the limited foreign currency earning options amidst continuing sanctions, Zimbabwe continues to prioritize tobacco [19]. This prioritization has been strengthened especially after the entry of China as a major investor [41]. China has invested significantly in Zimbabwe, from agriculture to construction, and remains Zimbabwe’s fourth largest trading partner in 2023 [42]. China also remains the largest buyer of tobacco from Zimbabwe [43].

Contract farming has strengthened the role of the private tobacco companies in Zimbabwe. Due to banks facing challenges following economic sanctions imposed on the country, farmers turned to contract companies for financial input for tobacco farming [24]. Contract companies actively recruit farmers to grow tobacco, provide input, financing and also buy the tobacco output from smallholder farmers [24]. In 2018, as per a Tobacco Industrial Marketing Board (TIMB) report, the private companies that had the largest market share in Zimbabwe were Zimbabwe Leaf Tobacco Company (13.2%), Northern Tobacco (12.2%), Mashonaland (11.7%), Premium (9.7%) and Tian Ze (9.2%) [44]. Despite its macro-economic importance, Zimbabwe’s tobacco farming sector is rife with many challenges. First, smallholder farmers face challenges in earning profits, forcing them into debt which follows the regional pattern in Africa of debt faced by tobacco smallholder farmers [2,3,5]. Second, the health of farmers is at risk due to tobacco farming practices [2,12,45]. A related concern is the use of child labor and the negative health impact on children who work on tobacco farms [46]. Third, Zimbabwe is increasingly losing its forests due to tobacco farming (for both farming land and curing of tobacco) [45,46]. The government and the tobacco industry in Zimbabwe have recognized deforestation as a key challenge and have launched reforestation programs, the impact of which requires further examination [45,46].

When considering domestic tobacco use, Zimbabwe’s demand reduction efforts—including regulating tobacco advertising—continue to face barriers due to tensions between tobacco control and the importance of tobacco as an economic commodity [3,9]. In 2021, the prevalence of tobacco use in Zimbabwe was 11.6%, with a much higher prevalence among men than women [9,47]. In 2019, 8% of all deaths in the country were attributed to tobacco use [9,47]. Zimbabwe is also facing a substance abuse crisis including the use of methamphetamine which disproportionately impacts youth. This crisis led the government to form an inter-ministerial task force to address substance abuse [48,49]. The association of substance abuse to tobacco use, especially among youth, is an important aspect that requires further examination.

Considering the definition of political economy note above, these contextual factors on wealth, power and politics, remain highly relevant to inform efforts to strengthen WHO FCTC implementation and support the development goals of Zimbabwe [14,16]. A crucial step in developing this understanding of political economy is soliciting perspectives of those working on topics directly or indirectly related to tobacco within the country. These perspectives can help illuminate the complexities of the political economy of tobacco, policy implementation, including supply and demand reduction, as well as other policy and market factors. To this end, we conducted a qualitative study that elicited the views of government, non-governmental, para-statal and other stakeholders in Zimbabwe. The objective of this study is to understand how key stakeholders in Zimbabwe view tobacco as an economic commodity and as a public health concern, including perspectives on alternatives to tobacco as an economic commodity.

## Methods

### Ethics statement

This study received ethics approval from the Institutional Review Boards of McGill University’s Faculty of Medicine and Health Sciences and the Research Council of Zimbabwe. For all interviewees in this study, we obtained written informed consent.

We used interpretive description (ID) methodology for this research as it allows us to gain diverse perspectives on complex topics that are difficult to capture using other methodologies [50]. According to Thorne et al., interpretive description “provides direction in the creation of an interpretive account that is generated on the basis of informed questioning, using techniques of reflective, critical examination, and which will ultimately guide and inform disciplinary thought in some manner,”[51]. While distinct in its approach, ID draws on well-established qualitative methods of data collection and analysis from grounded theory, naturalistic inquiry, and ethnography [51,52].

We employed purposive sampling to ensure diverse sectoral representation. We invited individuals who were involved in tobacco policy, including representatives from government, non-governmental organizations, parastatal organizations, unions, industry, and academic sectors. Across these sectors, our key informants (KIs) included subject matter experts and professionals in governance, environmental sustainability, business sustainability, business development, marketing, agriculture (both tobacco and non-tobacco), public health, and economic development. We conducted 23 key informant interviews (KIIs) between February 13, 2023, and March 31, 2023. The interviewers included SB, NN, AK, TN, and AC. We did not interview any minors – persons under 18 years of age as per Zimbabwe law [53]. Prior to the interview, interviewers explained the details of the project, reviewed the provisions of consent, described data collection and management of the project and the expected outputs. All participants provided written informed consent prior to the interview. Table 1 provides a summary of KII numbers associated with each sector. A semi-structured interview guide was developed based on a template that was built for KIIs in Mozambique and Zimbabwe (S1 Text) [54]. The interview guide aimed to explore the development goals of the country, the importance of tobacco to development, the impact of tobacco on health and wellbeing, and the progress of the implementation of the WHO FCTC including barriers faced and the future of tobacco farming in the country. Interviews ranged from 20-60 minutes with an average length of 30 minutes. One interviewee requested that the interview not be recorded due to the topic’s sensitive nature. In this case detailed notes were taken during and after the interview by the interviewers. All recorded interviews were transcribed verbatim, and interview data were anonymized then saved in a secure data platform.

**Table 1:** Key informant interviewees categorized by sector.

KII sector categories	KIIs (n = 23)
Governmental	6
Non-governmental organizations (NGO) and inter-governmental organizations (IGO)	6
Para-statal organizations	5
Tobacco industry	2
Academia	2
Farmer Unions	2

We used NVivo 14 software to organize the analysis and used an inductive analytical approach to identify themes that were relevant to the political economy of tobacco in Zimbabwe. Our analytical approach was two tiered. First, following a period of familiarization with the data, we initiated a process of iterative coding [55]. One researcher (SB) coded two complete interviews and generated an initial codebook. A second researcher (RL) checked for alignment of coding using the same two interviews. Once alignment was established via discussion, SB coded the rest of the interviews. This initial coding process served to organize excerpts from the transcripts according to child-codes and parent-codes (an aggregate of child codes). Second, we grouped parent codes into categories. When reading and coding the data, multiple aspects of tobacco in Zimbabwe including its relevance to the economy, livelihoods, health of the public, and health of farmers, were considered. Guided by ID methodology we used interview notes, coding memos, and literature (academic and grey) to further interpret the data and to collate data into six main themes for results reporting. Our interpretation of data including theme generation aimed to situate the data in the political economy of Zimbabwe. We used this approach to achieve ‘meaningful coherence’ from study design to data interpretation and reporting [56]. To provide a holistic picture of the political economy of tobacco and to situate interviewee quotes within the Zimbabwe context, we use official government data, data reported in the media from national government reports, data from inter-governmental organizations and research literature to complement the main emphasis on interviewee perspectives. This includes illustrating the points made by interviewees and providing necessary context for quotes from interviews.

Our positionality was informed by citizenship, research experience and lived experiences, which in turn influenced our analysis and reporting. Our authorship team consists of four Zimbabwean researchers living in Zimbabwe (AK, NN, TN, AC), four researchers in Canada (SB, RL, MH, AA) and one in the United States (JD). RL, AA, MH are Canadian citizens and JD is a dual Canadian-US citizen. SB is a Sri Lankan citizen and has over 5 years of policy research experience in global health. RL, JD have been working on the political economy of tobacco control co-led by colleagues based in the countries where the projects have focused (Kenya, Malawi, Zambia, Mozambique, Zimbabwe) for the past 15 years. MH and AA each have over 10 years of global health research experience. NN is an agronomist with over 10 years of experience in agriculture research and implementation of agricultural projects in the African region including Zimbabwe. AK has over 5 years of research experience in tobacco in Zimbabwe and over 10 years of agriculture and public health research in Zimbabwe. TN and AC are recently graduated undergraduate students in agricultural economics from University of Zimbabwe, Harare.

## Results

We developed six themes to illustrate interviewee perspectives on the key political economy features of tobacco in Zimbabwe: 1) macro-economic value and government prioritization of tobacco, 2) economic impact of tobacco on smallholder farmers, 3) tobacco and health, 4) deforestation and tobacco, 5) WHO FCTC implementation in Zimbabwe and 6) the future of tobacco in Zimbabwe. In Table 2 we summarize the main findings based on interview data and relevant contextual information. The following sections provide an in-depth report of findings, including conflicting interviewee perspectives and discrepancies between existing reports and interviewee perspectives.

**Table 2:** Main themes and key summary of findings per theme.

Main themes	Summary of findings per theme
Macro-economic value and government prioritization of tobacco	As indicated by all interviewees, tobacco remains a vital part of revenue generation at the macro-economic level for Zimbabwe. It is noted to be a key agricultural crop for foreign exchange earnings. Revenue from tobacco is highlighted as being essential for country’s economy. This is reflected in government prioritization of tobacco in policy.
Economic impact of tobacco on smallholder farmers	Farmers do not receive economic benefits from tobacco and are often trapped in debt cycles. Interviewees indicate that high priced inputs, unfair contracts, unstable tobacco prices, and existing debt affect profit margins. The contract structures and debt also affect smallholder famers’ ability to move away from tobacco.
Tobacco and health	*Considering tobacco use,* reports indicate there is a rising trend of tobacco use among youth. However, government and tobacco industry interviewees, supportive of tobacco farming have downplayed this impact. Public health sector interviewees (government, NGO, INGO) also highlight the need to collect more data, to make a compelling case. Public health sector interviewees also noted the rise in psychoactive drug use among youth. *Considering tobacco farming,* interviewees noted that farmers are severely negatively impacted due to lack of protective equipment, high labor demand for tobacco farming, and psychological impact of poor profits. Interviewees stress that better efforts are needed to improve farmer wellbeing.
Deforestation and tobacco	There is consensus across stakeholder groups that deforestation is a rising challenge cause by tobacco farming. Interview data indicate that while collaborative efforts between government and industry is underway to address this challenge – it remains a key concern. Some of the current efforts include planting gum trees, imposing levies, and supporting research on alternative energy sources.
WHO FCTC implementation in Zimbabwe	WHO FCTC implementation faces challenges due to government prioritization of tobacco as a key agricultural crop. Interviewees noted the difficult to make progress in supply reduction due to reluctance to reduce tobacco farming. In demand reduction, while some regulations are in place (e.g., taxes, health warnings on packaging), some interviewees noted the need for comprehensive efforts (e.g., reducing advertising). Interview data indicate that industry interference remains a key concern.
The future of tobacco in Zimbabwe.	Interviewees highlighted in the absence of strong economic alternatives to tobacco farming and better policy infrastructure to strengthen alternative crops, tobacco will remain a key agricultural crop for the foreseeable future. Interviewees noted that economic benefits of tobacco farming do not reach communities as expected.

### Macro-economic value and government prioritization of tobacco

The first theme includes the reasons for macro-economic prioritization of tobacco, the use of policy and the attitude of the government towards prioritizing tobacco.

All interviewees described tobacco as the foremost agricultural product of Zimbabwe. One government sector interviewee re-iterated the popular phrasing to highlight this prioritization, *“what do they call it? Zimbabwe’s golden leaf”* (anonymous interview, February 2023). All participant answers mentioned tobacco’s role as a foreign income earner at the macro-economic level. Government sector interviewees noted tobacco’s foreign exchange earning capacity for Zimbabwe as key factors for its attractiveness. As another government sector interviewee commented *“it’s the number one in terms of forex* (foreign exchange) *earnings right so we produce I think over 200 000 metric tons of tobacco…we view it as a 1 billion crop nationwide. It’s a 1 billion crop,”* (anonymous interview, February 2023).

**Fig 1:**
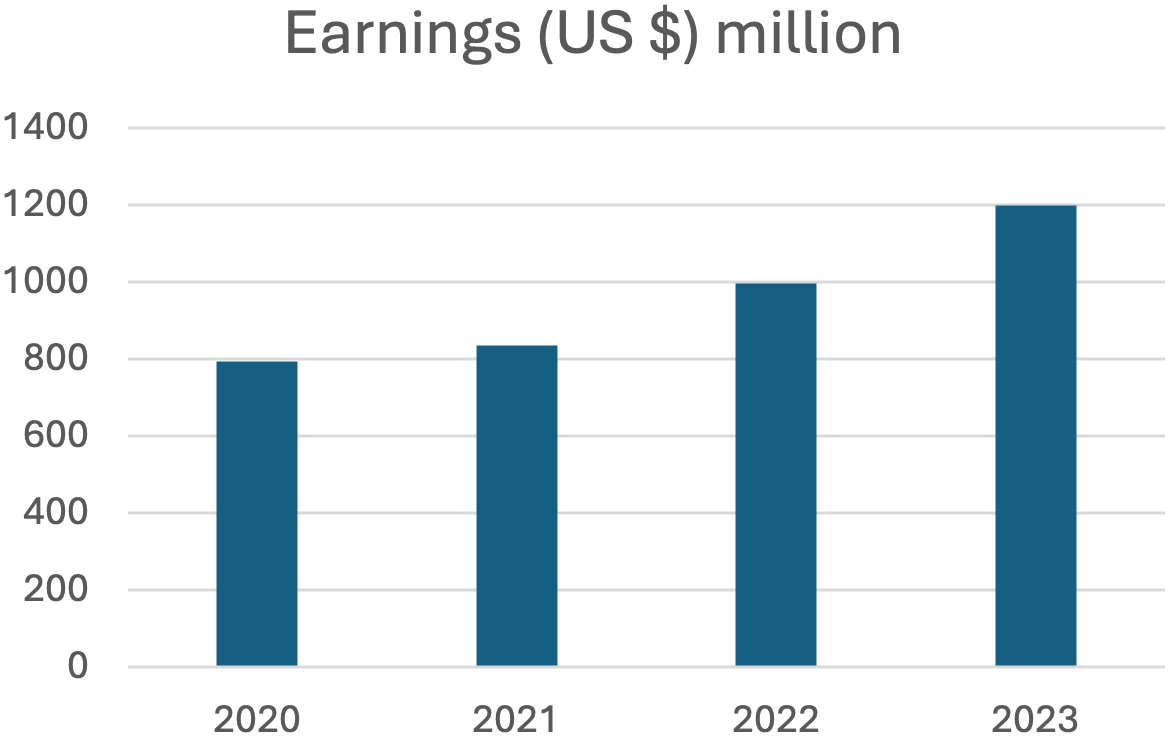
Total earnings from tobacco exports from 2020-2023 in Zimbabwe.

In 2023, Zimbabwe earned a reported 1.2 billion USD exporting 233,896 metric tons of tobacco leaf [18]. This is an increase in revenue of about 225 million USD over the corresponding period in 2022 during which Zimbabwe exported 196,565 metric tons of tobacco [18]. TIMB reports indicate as of October 27, 2023, tobacco cultivated area increased by 21% in 2023 compared to 2022 reaching a total of 19, 526 ha [57]. The average price for tobacco shipments was USD 5.26 per Kg in 2023 compared to USD 4.96 per Kg during the same period in 2022 [18]. As 2020 data indicates tobacco accounts for close to 17% of total exports and the absolute amount of tobacco exports has continued to increase since 2020 [8]. Fig 1 illustrates the increase of total earnings from 2020 to 2023 indicating an upward trend [58]. This increased output aligns with the Government of Zimbabwe’s development goal of strengthening agriculture as a revenue earner. This main goal includes inter-related sub-goals of reaching 300,000 metric tons of tobacco production by 2024–25 and using value added tobacco products to increase the value of the industry to US $ 5 billion by 2025 [19,36].

Availability of the international market, especially with the entry of China as a major investor, is another factor that interviewees highlighted. A government sector interviewee noted *“we’ve had quite a bit of approaches from the East, uh, China, the Middle East and so forth… So marketing (selling of tobacco) issues are not really a problem,”* (anonymous interview, February 2023). Zimbabwe and China signed a memorandum of understanding in 2005 and established the Tian Ze Tobacco Company (TZTC), a subsidiary of China Tobacco in Harare [41]. Preferential policy treatment such as the exemption of TZTC from the 2010 law that required all foreign owned companies to relinquish 51% of shares to local Zimbabweans, highlight the important role China plays in the Zimbabwean tobacco sector [41].

In addition to the government sector, interviewees from different sectors (e.g., para-statal, industry, farmer unions, NGO & IGO) also indicated the staying power of tobacco within the country’s economic agenda. One NGO sector interviewee noted this saying, *“You can come up with all sorts of perceptions, but I think Zimbabwe tobacco is here to stay. Whether people are lobbying against it, you know tobacco from Zimbabwe is highly sought after internationally,”* (anonymous interview, Febraury2023). Another NGO sector interviewee echoed the industry narrative [3,5] of tobacco’s value for poverty eradication and a pathway to achieve Sustainable Development Goals (SDGs) 1 and 2 dedicated to eradicating poverty and hunger:

*“…so I believe as a country, we still need the production of tobacco in order to eradicate SDG1 and SDG2, but while we do that, we have to put in controls, policies that ensure that consumers, especially the youth, are not exploited”* (anonymous interview, March 2023)

Tobacco production in Zimbabwe is a multi-stakeholder effort with coordination at the center. Interviewees pointed out that government efforts are directed to supporting tobacco as an economic commodity through inter-ministerial, industry and parastatal organizational arrangements dedicated to tobacco supply. Most of the interviewees singled out the parastatal arm, the TIMB, as the organization responsible for convening all tobacco production related stakeholders, government tobacco production policy implementation and regulatory action. In addition to TIMB, participants delineated farmer unions, tobacco associations, contracting companies, auction floor operators, government ministries, and the Tobacco Research Board as key players. Moving beyond those focused on agriculture, finance and trade, interviewees also highlighted the Forestry Commission and other environmental agencies as relevant to tobacco farming due to partnerships focused on reducing deforestation. Tobacco industry-backed groups such as the Sustainable Afforestation Association work with the government and smallholder farmers to address the deforestation challenge posed by tobacco farming. Government stakeholders stressed the fact that government aims to work with these groups, facilitating collaboration. A government sector interviewee noted this saying *that “…we are really encouraging that we work together. So, government is not competing with private sector, but we complement where the private sector or NGO is doing their work, uh, in the areas we don’t go there and do the same work,”* (anonymous interview, February 2023). As per government sector interviewees, the Zimbabwean government continues to prioritize tobacco, embedding strategies to enhance the tobacco production sector within national development goals with TIMB (parastatal) as the main operationalizing organization.

### Economic impact of tobacco on smallholder farmers

Under the second theme we illustrate the conflicting perspectives on tobacco as a commodity beneficial for smallholder farmers. Despite the emphasis put on tobacco as an important economic commodity for the country, the majority of the interviewees highlighted how smallholder farmers are unable to turn a profit from tobacco farming. This pattern has also been identified in Zimbabwe where tobacco farming has caused smallholder farmers to become trapped in debt cycles with contracting companies [5,59]. Interviewees from parastatal, farmer union, government sectors indicated high prices of inputs, fluctuating pricing of tobacco and weather conditions as major factors impacting profit. One para-statal sector interviewee noted that *“Farmers are finding it difficult to make a living out of this whole tobacco production because of the highly priced inputs and what they will then realize on the market, it’s not really breaking even,” (anonymous interview, February 2023).* An industry sector interviewee noted the presence of a “false profit” from tobacco earnings saying, *“the family members to do the farming... So, when calculating, after selling, they don’t deduct the cost of production from the revenue which they make. So, I’m saying most of them, they break even” (anonymous interview, March 2023).*

Interviewees also highlighted that the profits from tobacco farming usually do not end up with farmers but with middlemen such as leaf buyers and those who manage the buying process. An academic research sector interviewee noted that the buyers and processors “*are the guys who are really getting much of the marketing margins in tobacco,” (anonymous interview, March 2023).*

There were counter narratives, especially from the government sector. Some government sector interviewees highlighted how tobacco remains a key strategic crop in alleviating rural poverty for the government and that some farmers have dedicated more land to tobacco. These interviewees, based on the above claims, suggested that tobacco farming is profitable for smallholder farmers. Interviewees also indicated that one of the many reasons for tobacco farmers to continue growing tobacco is that they lack alternatives for a cash crop with an established market value chain. The structure of the contracts and particularly the annual repayment of loans was noted as a challenge: *“If we had long term contracts, like previously on commercial farms, a farmer could get like a long-term contract of 10 to 25 years, now with these small-scale farmers, the maximum is one year, so recovering everything in one year is very difficult,”* (anonymous interview, March 2023).

In summary some interviewees identification of the lack of profit for smallholder farmers which traps them in debt with private contracting companies is at odds with the high foreign exchange earning potential noted by interviewees at the macro-economic level.

### Tobacco and health

Within the third theme, we examined health in Zimbabwe as it pertains to tobacco from two angles: the health of the public related to tobacco consumption and the health of tobacco farmers.

### Tobacco use in Zimbabwe

One of the recurring points raised by both industry and government interviewees supportive of tobacco farming is that there is a low recorded consumption rate of tobacco in Zimbabwe, despite being a key exporter of tobacco. However, according to WHO, age-standardized estimated tobacco use prevalence in 2021 was 21.9% among men and 1.2% among women, with 11.6% for both sexes [9]. In 2015, Demographic Health Survey (DHS) recorded 17.7% of men and 0.5% of women aged 15–54 smoked tobacco. The 2014 Global Youth Tobacco Survey indicated 20% of all youth aged 13–15 use tobacco (smoking and smokeless). These prevalence rates are in the middle of the range based on African region estimates for tobacco use. That youth prevalence rates are higher than current adult rates indicate a problematic trend toward increased tobacco use in the future. The regional estimates indicate a range of 4.6% to 36.6% for adolescent girls and 7.8% to 36.5% for boys as per WHO African Region Office [60]. Zimbabwe’s prevalence monitoring also requires strengthening with the last countrywide survey completed in 2015. As of December 31, 2022, the National Tobacco Control Program had one full-time staff member [9]. This challenge of monitoring tobacco use was noted by interviewees from the health sector representing both governmental and NGOs. A government sector interviewee noted this saying, *“young people, they use tobacco, but I don’t have any statistics to quantify how much of tobacco use is happening,”* (anonymous interview, March 2023). Despite evidence to the contrary, most interviewees including the health sector experts in governmental, non-governmental and inter-governmental sectors repeated the notion that tobacco prevalence is observed to be low [9]. One academic sector interviewee from the health sector noted that there is a preference among youth for psychoactive drugs saying,

*“So, I think that’s the issue now with tobacco, that, you know, it doesn’t give a high that lasts. So, our young people are now looking for alternatives. So, we are not likely going to see challenges with tobacco in the near future, but our main challenges are going to be in the area of hard drugs,”* (anonymous interview, March 2023).

A non-governmental sector interviewee focusing on health noted that alcohol and tobacco use can be associated with psychoactive drugs but that more research is required to ascertain this link, saying, *“But they start with tobacco. It’s just cigarettes and alcohol. Then maybe later they graduate to those dangerous substances,”* (anonymous interview, February 2023). Given the high tobacco use among youth, some health focused non-governmental interviewees, indicated that there is a need for better coordination among public health actors and targeted advocacy for demand reduction policies.

### Tobacco farmers’ health

All interviewees who indicated that they had knowledge on tobacco farmer health highlighted that this was an urgent and important concern. Similar to what has been observed in the region, many interviewees pointed to the handling of chemicals, inadequate protective equipment, being continuously exposed to tobacco and harmful chemicals inhaled during tobacco curing as key risk factors related to farmer health [61]. The release of *Bitter Harvest*, a report by the Human Rights Watch in 2018 put the spotlight on farmer health and wellbeing including the use of child labor and exposure of children to harmful chemicals in tobacco farms in Zimbabwe [46]. The interviewees that focused on farmer wellbeing also highlighted the detrimental mental health implications of unprofitable tobacco growing within a broader context of economic hardship including high levels of inflation. As per interviewees the challenging socio-economic conditions due to the unprofitability in smallholder farming in tobacco (and even in other sectors) can lead to self-harm. One academic research sector interviewee working in public health noted this saying,

*“But in terms of the health and well-being of farmers, I think contract farming has really been detrimental. So, I think we can talk about issues to do with mental health, because many times farmers are not able to actually reach the projected target of output…And then they use those pesticides, which they are given as part of the inputs, then they use those pesticides to commit suicide,”* (anonymous interview, March 2023).

Following the increased spotlight on working conditions and child labor, the government and para-statal stakeholders have focused more on requiring industry to provide personal protective equipment (PPE) and further training to farmers. Interviewees indicated that while harm reduction and building awareness on tobacco use is a health sector responsibility, the responsibility of protecting farmer health remains with Ministry of Lands, Agriculture, Fisheries and Rural Development and other tobacco related stakeholders. One IGO sector interviewee pointed out that the government is already aware due to multiple health focused stakeholders raising this issue within the government.

*“Yeah, it’s something that is also recognized as health problem… There are various communications and discussions that they carry out with farmers, of course, in association with Minister of Health and Child Care in terms of educating farmers about the health risks of, uh, pesticides that are used in agriculture and how they can sufficiently protect themselves from those, uh, using, ah, appropriate protective, uh, uh, clothing,”* (anonymous interview, March 2023).

Para-statal interviewees indicated that they have taken significant measures to protect farmers including requiring contractors to provide necessary PPEs. One para-statal sector interviewee noted this saying *“Someone has to take responsibility. So, it’s part of the compliance framework. You provide the inputs, you provide the chemicals, provide training and also provide protective clothing.”* (anonymous interview, February 2023). However, interviewees researching and working on farmer wellbeing challenge the idea that there are sufficient efforts to address farmer health and wellbeing by the government and related tobacco farming stakeholders. Some interviewees also question the commitment of tobacco contracting companies to ensure farmer wellbeing. One academic sector interviewer highlighted that tobacco smallholder farmers not having the status of contract company employees is a loophole that has been exploited: “*And one argument that I heard is that it’s (not providing PPEs) because they said farmers are not their employees, so farmers need to care for their own health,” (anonymous interview, March 2023).*

Tobacco farming sector related (including farmer unions and NGOs) interviewees highlighted that there are increased efforts to remove child labor from tobacco farming. They highlighted that attention on child labor in tobacco farming has created negative publicity for the tobacco industry, which can lower sales. This decrease in sales remains a key motivational factor for the industry to control child labor. One para-statal interviewee highlighted that the government bodies have already formed working groups to address child labor and other human rights challenges in tobacco.

*“We’ve been working with the Ministry of Public Service, Labor and Social Welfare, we’ve been working with the grower representatives themselves, they are part of the working group that is in place to make sure that we address child labor issues and human and labor rights issues. You know there are so many other organizations that are on the lookout, they just watch, they observe it, whatever is happening, and any negative publicity will then affect our industry,” (anonymous interview, February 2023).*

In summary, based on KIIs, there was consensus on negative impacts of tobacco farming on farmer health and the importance of ensuring farmer health and wellbeing. While government and industry highlight policies aimed at safeguarding farmer health, success of these policies at the implementation level remains uncertain.

### Deforestation and tobacco farming

Interviewees consistently highlighted the prominence of deforestation due to tobacco farming as a major concern within Zimbabwe and discussed opportunities and challenges facing ongoing solutions.

Zimbabwe is facing a significant challenge due to deforestation related to tobacco farming [62]. One para-statal interviewee pointed this out saying *“Zimbabwe, we are losing about 262,000 hectares of forest every year….the tobacco farming. It’s (tobacco) also contributable (contributes to) to 20% of that chunk,”(anonymous interview, February 2023)*. A report produced by the Government of Zimbabwe entitled Zimbabwe’s Nationally Determined Contributions to the Paris Agreement highlights firewood sourcing, settlements, agricultural activity, wildfire, tobacco curing, charcoal sourcing, brick making, logging, overstocking, construction, mining and brushwood sourcing as direct drivers of deforestation in Zimbabwe [63].

Loss of indigenous forests remain a key concern for both government and industry stakeholders. Industry associated interviewees highlighted the economic impact of deforestation on tobacco as a key concern and along with several interviewees from different sectors contrasted the current experience in Zimbabwe with Malawi. One NGO sector interviewee highlighted an ominous pattern, noting that:

*“If we look at tobacco production, Zimbabwe used to compete against Malawi on Virginia tobacco production. And now Malawi has stopped, literally stopped producing Virginia tobacco. Why? Because they finished all their trees. Yes. We want to continue supporting government in terms of production of tobacco, Virginia tobacco, because it is, tobacco comes as number one in the agriculture basket,” (anonymous interview, February 2023).*

Many interviewees, while highlighting the economic value of tobacco, were concerned about the lasting impact of pursuing revenue at the expense of forests. One parastatal sector interviewee, after highlighting 90% of tobacco farmers are reliant on indigenous forests, observed the lasting impact of deforestation saying,

*“That (is) what we are really exporting, it’s not tobacco (as) they say, but we are exporting our forest. When we say this year, we are targeting 300 million kgs of tobacco, it’s achievable, but it’s coming at the cost of our forest,” (anonymous interview, February 2023)*

An NGO sector interviewee highlighted the challenge of the increasing levels of wood required to cure tobacco saying,

*“What are the implications? One kg of tobacco requires about 10 kgs of wood to cure it. Which means the minister is saying if we are to sustainably cure that whole crop, we need about 3 billion kgs of wood. That’s a huge volume of wood,” (anonymous interview, February 2023).*

As government, parastatal, industry and NGO sector interviewees indicated, the government and industry are collaborating on planting trees (mostly gum trees – e.g., eucalyptus) for the use of tobacco curing. The Forest Act, the Communal Lands Forest Produce Act, Environmental Management Act, Parks and Wildlife Act, Rural District Councils Act, Statutory Instrument 116 are some of the key legal instruments for protecting forests [63]. Many interviewees (parastatal, NGO, industry) point to policy measures such as the afforestation levy that is being collected by the government to support afforestation in Zimbabwe. However, the distribution of the funds to relevant stakeholders such as the Forestry Commission has been slow. A parastatal sector interviewee indicated that the 1.5% levy deducted from gross sales of tobacco starting in 2015 was only channeled to relevant stakeholders from the treasury in 2019. Interviewees expressed that even when levies are collected properly on the auction floor, it takes time to reach the afforestation stakeholders from the government treasury and only a “trickle” of the funds, reach them.

Despite existing challenges on levies noted above, Industry interviewees expressed that there is concerted collaborative effort put forth by the government, para-statal organizations along with the tobacco industry to mitigate deforestation. One industry sector interviewee noted this saying,

*“We are getting massive support from the government and the para state, like Forestry Commission. They are assisting us very well. We as a company also take part in the government programs for tree planting. You know, Forestry Commission is a regulator of forestry in Zimbabwe. When we face challenges (related to afforestation) with the communities we work with, if we engage them (Forestry Commission), they quickly assist us,” (anonymous interview, March 2023).*

Stakeholders from both the government and industry noted the challenge of not having alternative sources of energy to cure tobacco. Interviewees from parastatal, government, NGO and industry sectors indicated that while coal is an option, as it is a non-renewable source of energy, its use is being discouraged due to global agreements on climate crisis. Solar energy has not been successful either, with solar powered curing barns too expensive for smallholder farmers [64].

Interviewees noted that the key stakeholders related to afforestation efforts include Environmental Management Agency, Forestry Commission, industry backed Sustainable Afforestation Association, contracting companies, and para-statal bodies. Additionally, research institutes such as the Tobacco Research Board also contribute via research for alternative sources and increasing energy efficiency for tobacco curing. Yet, these efforts largely remain a work in progress without immediate solutions for forest coverage depletion in Zimbabwe. A parastatal sector interviewee summarized this saying,

*“So, it’s one big challenge. As much as we want to address deforestation challenges, we need to make sure that we avail an alternative that is affordable. So, it’s a challenge. We are working with the industry, we are working with other researchers to try and see how best we can come in and help out the growth, yeah,” (anonymous interview, March 2023).*

While addressing deforestation seems to be a priority on the government agenda, interviewees suggested that it is also important to examine whether the cost of protecting Zimbabwe’s forests and increasing tobacco output is being transferred to smallholder farmers. One industry sector interviewee pointed this out saying,

*“I feel it (deforestation) was not looked into for years, sometimes, but if what we are doing now, if it was done like 20 years back, I think it will be at around 100% sustainable….For now, yes, we are waiting for the trees which are growing to mature in other areas like Manikaland area, we provide firewood, but areas like Mashonaland waste, Mashonaland central, it is very far and the transport cost is very high. If we charge the farmer, say 20 cubes of firewood, if we charge him, he will remain with nothing in his or her pocket,” (anonymous interview, March 2023).*

The *Drivers of deforestation and forest degradation in Zimbabwe (2019)* report produced by the Government of Zimbabwe identifies lack of sustainable resources, issues of governance and political interference as the main challenges to sustain work related to countering deforestation [63].

As indicated in this section via KIIs, prioritization of efforts to address deforestation by government and industry has led to forming inter-sectoral coalitions. However, lack of alternative energy sources (despite ongoing government sponsored research) and the government targets of increasing tobacco production output leaves the deforestation challenge in Zimbabwe unresolved.

### WHO FCTC implementation status

The fifth theme focuses on the challenges and opportunities of WHO FCTC implementation in Zimbabwe. Zimbabwe acceded to the WHO FCTC in 2014 [10]. At the time, critics noted that acceding to the WHO FCTC will not soften the anti-tobacco control stance of the country but suggested it may serve as an opportunity for the government to prioritize public health [10]. Additionally, health focused SDG 3 (specifically target 3.a) includes targets for all countries to implement WHO FCTC [65]. There is consensus across all interviewees that effective policies and implementation for supply reduction is at least 20 years away in Zimbabwe. Interviewees from across sectors provided various reasons including foreign currency earning potential, the pride the government (and Zimbabweans) have in its agricultural sector, existence of a strong market especially with China as an investor, lack of a competitive alternative to tobacco and the prevailing poverty levels. These prevailing notions are also affirmed by the government’s strategic development plans that aim to continue tobacco as a key crop [19]. Currently, Statutory instrument 264 of 2002 remains the government tobacco control regulation used to implement the WHO FCTC especially for demand reduction [66].

Many interviewees (government, parastatal, academic, NGO) highlighted conflict of interest within the government coupled with strong industry presence makes WHO FCTC implementation processes difficult. A government sector interviewee pointed out that to even attempt to talk about harm caused by tobacco farming or supply reduction policy implementation is a daunting task. Therefore, many health sector stakeholders focus on demand reduction.

*“In terms of tobacco growing, I think there’s little you can do about it… Also, it depends on the finances which are obtained from the tobacco growing. So, what you need to really talk about, as I said before, (is) more of a reduction of smoking. Because growing they always talk about it, is part of economic, it brings economic growth and the people are tobacco growers,” (anonymous interview, March 2023).*

Currently, complete smoke free laws are applied to healthcare facilities, public transport, universities, educational facilities other than universities, theaters, museums, youth centers, places of worship and public meeting halls [67]. However, this leaves out important places such as government facilities, indoor offices and workplaces, restaurants, cafes, pubs, and bars which can have designated smoking areas [67]. Even in places where smoking bans are required, the minister has the power to make an exception and allow people to smoke [9].

One IGO sector interviewee pointed out mixed progress in terms of labeling and advertising. Zimbabwe does not have any advertising restrictions and allows companies to sponsor sports teams and advertise on any platform [9]. Advertising and sponsorships are key strategies that the tobacco industry uses to gain young consumers [68]. WHO FCTC requires member states to comprehensively ban tobacco advertising, promotion and sponsorship.

*“…in terms of labelling for tobacco products, the country requires that the contents of tobacco like tar, nicotine, be well labelled, and that is (on)going. And the issue that tobacco is hazardous is also labelled on all tobacco products, at least those which are formally marketed. And there are other issues, of course, which we are still to work on, have not yet discussed… the issues of plain packaging, which actually the FCTC is promoting… the advertisement both on the electronic media and on the packages is actually going on,” (anonymous interview, March 2023).*

Additionally, while Zimbabwe has continued to raise taxes on manufactured tobacco products, governmental, NGO and IGO interviewees point out that tobacco taxes are not sufficient, and that tobacco products remain cheap in Zimbabwe. Therefore, interviewees noted that price is not a deterrent to tobacco consumption. A government sector interviewee highlighted this saying, *“the Ministry of Finance… they raise taxes every year so there were raising (of taxes)...but it was insignificant,” (anonymous interview, March 2023).*

*An IGO sector interviewee also supported this claim saying,*

*“Tobacco in Zimbabwe is not expensive. It’s actually cheap. What we would have recommended the government is to introduce more prohibiting costs, uh, for, uh, tobacco, but which the government is not yet ready for that, at least as far as I know now. Probably because of, uh, the economic situation, some members of the society may not, uh, be able to buy the filter cigarettes, but they are able to buy other forms of cigarettes.” (anonymous interview, March 2023).*

Zimbabwe scored 1.25 (from 0-5) in the 2021 Tobacconomics Cigarette Tax Scorecard in the Tobacco Atlas [47]. Tax related policy implementation is low compared even to the African region, which is the lowest among WHO regions, and more so to the rest of the world [47]. The tax scorecard measures price, change in affordability, tax share, and tax structure, and Zimbabwe fares poorly on all four.

Industry interference – or in Zimbabwe’s case industry’s direct access to the policy makers and ministers - makes it difficult for relevant government officials to implement key provisions of the WHO FCTC. For example, a government sector interviewee highlighted that if there is a policy implementation effort that the industry did not prefer (such as advertising restrictions) they would directly call the minister responsible which would in turn require an explanation from those ministry staff members implementing the WHO FCTC.

Zimbabwe’s health sector actors have been trying to implement at least some of the key demand reduction measures of the WHO FCTC via the Statutory Instrument 264 of 2002. A government sector interviewee cited lack of resources as a key challenge:

*“But the main problem is we didn’t have enough resources really. Even though we were being helped by the WHO on days like you know, international, no smoking day, we trying to inform people of the dangers of smoking… Come up on the TV, write the newspapers, come up with the pamphlets, and try to distribute it throughout the provinces but the problem was the resources.” (anonymous interview, March 2023).*

Both governmental and non-governmental stakeholders interviewed indicated lack of resources as a key limitation to WHO FCTC implementation. Inability to advocate for supply reduction policy due to tobacco being a key economic commodity is another key barrier as per interviewees. Additionally, interviewees noted the need for tobacco control focused research in Zimbabwe to use in advocacy, strategic alliances among national level and intergovernmental health focused stakeholders and improving communication and coalition building to advocate at the highest levels of the government.

### The future of tobacco in Zimbabwe

Under the final theme we report interviewee views on the future of tobacco in Zimbabwe with the existing tensions between WHO FCTC implementation and government prioritization of tobacco production.

Government, industry, and tobacco-focused parastatal organizations are well aware of the WHO FCTC and country-level responsibility. Government sector interviewees noted that Zimbabwe is moving towards tobacco processing and producing value added tobacco products as viable strategic plans. This shift is also in line with statements from the Ministry of Agriculture. One government sector interviewee mentioned that there are preparations for the potential success of the “anti-tobacco” campaigns which may lead to low global demand, which in turn will negatively impact Zimbabwe’s economy. Some of the alternatives to tobacco mentioned by interviewees included horticulture including fruit crops (mangoes, blueberries) and industrial hemp *(anonymous interview, February 2023)*. Research by the Zimbabwe Economic Policy Analysis and Research Institute also indicate that farmers mention soya, cotton, ground nuts, and sunflower as other plausible alternatives [69].

Another government sector interviewee highlighted strategic shifts are needed within the government to keep the money from tobacco production moving out of the country.

*“It’s a 1 billion crop but the benefit to the nation, the benefit to the country, the communities and the livelihoods of our people has been very very low. It was literally the funding structure - that it has been funded from offshore….I think we would probably get around 200 million as a country from 1 billion,” (anonymous interview, February 2023).*

Recent research from Zimbabwe indicates that farmers are willing to switch to alternatives if there is adequate profit and dependence on tobacco can be broken with key policies that support alternative crops [69]. Such policies include providing capacity training for growing alternative crops including extension workers to support farmers, strengthening research into alternative crops and strengthening farming models for them [69]. Therefore, the policy infrastructure plays a significant role in deciding the longevity of tobacco in Zimbabwe as an agricultural crop. A parastatal sector interviewee with economic expertise pointed this out saying,

*“Yes, the market is a factor, but I think market also can be propped up by policy. If the other crops are also highly paying like tobacco, you can see a shift in that area. If you only look at tobacco is also very labor intensive. It is almost 9 months of preparing. But the other crops (are) more like three months production cycle which can equally be productive with high returns,” (anonymous interview, March 2023).*

Government sector interviewees highlighted that political will remains central to whether or not tobacco will continue to be a key crop and whether the WHO FCTC will be successfully implemented. One of the government sector interviewees noted this saying, *“Let’s be honest. It’s going to be challenging. Unless the government looks for viable alternatives for the farmers, then, yes, you can now talk about that framework convention,” (anonymous interview, February 2023).*

Based on KIIs, perceived economic benefits remain a central consideration within the decision-making processes of the government, although some within the government recognize that communities do not necessarily economically benefit from tobacco. Interviewees with economic expertise indicated that creating a better policy environment to facilitate alternative crop production can help smallholder farmers transition from tobacco. While alternatives to tobacco have been considered to an extent, prioritizing alternative crops over tobacco remains unlikely for at least a decade in Zimbabwe.

## Discussion

The political economy of tobacco in Zimbabwe has clear implications for the success of WHO FCTC implementation. Political economy factors found to be important include sanctions imposed on Zimbabwe [70,71] government developmental priorities [65,72], availability of markets for alternative crops [5,24], and the impact of the tobacco industry [24,41,73] among others. Our findings brought together diverse stakeholder perspectives and relevant contextual information to examine the political economy of tobacco in Zimbabwe including the status quo of WHO FCTC implementation and future outlook. The six main themes of our findings include macro-economic value and government prioritization of tobacco, economic impact of tobacco on smallholder farmers, tobacco and health, deforestation and tobacco, WHO FCTC implementation in Zimbabwe and the future of tobacco in Zimbabwe. Drawing from these findings, we see three key policy opportunities relevant to both economic and public health priorities in Zimbabwe: improving public health via demand reduction, prioritizing smallholder farmer wellbeing and addressing deforestation challenges related to tobacco. All three opportunities signal a need for the Zimbabwean government to strongly consider better implementation of the WHO FCTC to achieve demand and supply reduction goals.

First, in terms of public health, the oft repeated argument of ‘we export but we do not consume’ especially by government interviewees needs re-examination. As survey data and projected estimates indicate, Zimbabwe has a relatively higher prevalence of tobacco use among men and adolescents [9]. As the WHO African regional office indicates, tobacco use among adolescent boys and girls are on the rise in the region, dispelling the common narrative that tobacco use is only high among adolescent boys [60]. Furthermore, research indicates that the susceptibility to tobacco use among adolescents who have never used tobacco before is also high in Zimbabwe [74]. Thus, the available prevalence data on tobacco use in Zimbabwe signals ongoing and worsening public health challenges related to tobacco. Additionally, as interviewees indicated, Zimbabwe is also facing unprecedented challenges of substance abuse among youth, leading to significant physical and mental health challenges [75,76]. Research has indicated that tobacco use is associated with future substance abuse among youth [77,78]. The current policy infrastructure in Zimbabwe allows tobacco advertising and sponsorships targeting youth [9,68]. The formation of an inter-ministerial task force and intentionality displayed from the President’s office downwards shows that improving the health of youth remains a clear government priority [1,9,76]. Therefore, a potential opportunity for the government is to realistically evaluate and consider the impact of tobacco among youth (and in the general population). This consideration can be supported by addressing some of the challenges noted by interviewees. Viable approaches include assessing and updating prevalence data on tobacco use, increasing WHO FCTC implementation staff (currently one staff member) [9] and safeguarding public health sector staff from industry interference. Given that the government is urgently concerned about the wellbeing of youth, in addition to substance abuse reduction efforts, a policy window is open for the government to consider the necessity of tobacco demand reduction efforts such as regulating marketing, introducing plain packaging, and effectively addressing other tobacco sales efforts targeting youth. Zimbabwe can also learn from policy efforts in regional peer countries such as smoke-free environments and effective taxation policies in Nigeria and South Africa to reduce tobacco consumption [79].

Second, farmer wellbeing is impacted by tobacco. There is a direct negative impact of tobacco farming on health of farmers (and their families) as noted in literature, which was also stressed by interviewees [2,46,80]. This direct impact is worsened by the lack of income for tobacco smallholder farmers or, as one industry sector interviewee indicated, a ‘false profit’. Contract farming mechanisms often lead to farmers being trapped in debt cycles as has been evidenced in Malawi, Kenya, Mozambique, and Zambia, and countries in other regions of the world such as Indonesia and the Philippines [81–84]. [5,81–84]. As indicated by interviewees and intergovernmental organizational reports, food security is also a concern and a key priority in Zimbabwe [39,80]. Given the multi-faceted impacts on the population, political will and novel arrangements are necessary to shift complex agricultural value chains and prioritize wellbeing of farmers and food production [72]. As many interviewees pointed out and as government strategy documents indicate, the complete shift from tobacco will take time [19,65]. Yet, Zimbabwe’s development strategies can consider incremental policy efforts [85,86]. Zimbabwe can draw from policy efforts in Zambia and Kenya that supported small groups of farmers to successfully transition to alternative crops [85,86]. In Kenya, the government, in partnership with WHO, the World Food Program (WFP), and the Food and Agriculture Organization to support tobacco farmers to switch from tobacco to high iron beans, including input support and a ready market via WFP’s local procurement initiative [87].

Finally, as interviewees, government reports and research literature indicate, Zimbabwe is facing a significant environmental challenge of deforestation due to the high demand for wood to support tobacco curing [46,63,64]. Government and industry coalitions have tried to address this challenge but, as interviewees have indicated, these efforts are not sufficient. Interviewees from Zimbabwe identified Malawi as a cautionary tale of deforestation effects due to tobacco farming. However, Zimbabwe too is facing this rising tension of wanting to meet higher tobacco production targets yet safeguard its forests [63,88]. The government announcement in 2021 of the Tobacco Input Revolving Fund of US $ 60 million aimed at providing funds from local sources for tobacco was welcomed by industry stakeholders [89]. This fund (yet to be released) is in line with the government’s strategic development goal of making tobacco a US $ 5 billion (with 300 million Kg production per year) crop by 2025 [89,90]. While the minister of agriculture has indicated the target will be reached by post-harvest minimization of loss, as per many interviewees and government reports, deforestation remains a concern [62,63,90]. Reaching a higher tobacco output target will also require more energy to cure tobacco and alternative fuels have failed to take hold [64]. In Zimbabwe 62% of Virginia tobacco produced is cured using wood [64].

It is necessary to reassess if policy tools aimed at addressing deforestation such as afforestation levies are effective and whether these levies transfer the burden to already impoverished tobacco farmers. As interviewees have indicated and news outlets have reported, there have also been delays and challenges for the funds to reach relevant afforestation stakeholders such as the Forestry Commission [91]. Furthermore, many interviewees expressed concerns about insufficient income for farmers. It will be important to consider if added levies will affect the well-being of the farmer by impacting their income. Tobacco farmers’ support of the levy is reported to be low with farmers arguing that they are not benefiting from the fund [91,92]. Only 4500ha of gum trees were reported to have been planted by February 2024 [91]. Farmers worry that the slow pace of the initiative could jeopardize Zimbabwe’s future access to the European Union (EU) market due to EU’s new deforestation related regulations on import commodities [77].

Given the multi-pronged sustainability challenges that the tobacco industry poses, including its heavy environmental cost, strengthening government support for alternative crops could be a potential solution for the government to explore with medium to long term benefits in mind [80]. From a policy and technical support perspective, WHO FCTC Article 17 can provide policy implementation pathways for the government. Article 17, with its focus on viable alternatives, can support the government’s existing considerations to diversifying its agricultural product range and protecting its environment [64,93]. In 2023, WHO FCTC released a toolkit for governments and other stakeholders to use to explore policy pathways to strengthen alternative crop production [94]. This toolkit provides resources and clear pathways for the following: situational analysis, understanding factors required to facilitate alternatives, understanding sectoral contributions to implement Article 17, policy options and mechanisms of support for alternative crop production and managing industry interference, [95]. It also includes a tool for measuring progress of change so that governments can assess the success of their policies in diversifying away from tobacco [95]. As per our study participants, Zimbabwe has already considered diverse agricultural options. The 2022 TIMB strategic plan includes tobacco farmer crop diversification as one of its focal areas, aiming to increase the share of farmer income from alternative crops from 5% in 2022 to 25% in 2025 [96]. Therefore, Zimbabwe can further strengthen its efforts in crop diversification in a manner that complements national development plans. It will not be uncharted territory as Zimbabwe can learn from best practices of other countries (Kenya, Zambia) in the region and use available tools provided by WHO FCTC (Article 17 toolkit) to realistically achieve this stated goal.

## Limitations

The main limitation of our study is the low number of participants from the tobacco industry. One potential reason for this is industry viewing academic researchers as biased against the tobacco industry. Some potential participants declined our invitation for interviews, given the sensitive nature of the topic and tobacco being a high government priority. Topics related to tobacco can be considered sensitive in Zimbabwe by many due to tobacco farming being a highly politicized topic and an economic priority. Additionally, the interviews were conducted close to a presidential election which may have also affected the inclination to participate given the political nature of the topic. As noted, we also had one participant request not to be recorded even with the knowledge that the data will be anonymized. Given these potential limitations, we chose to embed reference to document sources throughout the results section. We thought that having comparisons between interviewee perspectives and existing analyses on topics that they discussed would add a layer of objectivity to our findings.

## Conclusion

Amidst a complex political economic context, including the historical entrenchment of tobacco growing within the country, the post-land reform shift to smallholder farming as a strategy to strengthen rural economies, and a tumultuous political environment that has led to the imposition of sanctions by several countries, Zimbabwe continues to prioritize tobacco as a key revenue earner. The relationship between tobacco as an economic commodity and limited commitment to tobacco control is apparent. Tobacco continues to pose significant challenges to smallholder farmer wellbeing. Tobacco farming has an impact on tobacco demand reduction efforts and comes with a heavy environmental cost. The current challenges offer the Zimbabwean government an opportunity to pivot away from tobacco and seriously consider alternative options, in order to improve its agricultural economy and protect the wellbeing of its population. It is also vital that global actors recognize the impact of longstanding sanctions and restrictions on Zimbabwe’s political economy. Especially, how sanctions may be impeding the development of markets that lessen the importance of tobacco in terms of export earnings.

## Supporting information

S1 Text Semi-structured qualitative interview guide. (DOCX)

S2 Text PLOS’ questionnaire on inclusivity in global research (as requested by the journal). (DOCX)
