# Supplementary material for: The political economy of tobacco of Zimbabwe: An analysis of stakeholder perspectives
Source: PLOS Glob Public Health. 2025 Jun 30;5(6):e0004805. doi: 10.1371/journal.pgph.0004805 (PMC12208465; doi:10.1371/journal.pgph.0004805)
Supplement: S1 Text — (DOCX) [file pgph.0004805.s001.docx]

**Supplementary File 1: Key Informant Interview Guide - Zimbabwe**

**This is a semi-structured interview guide and all questions will not be used. The interview will be guided by the answers of the interviewees.**

**The identity of the interviewees or organization or ministry will not be used in data analysis or in publications.**

Generic questions, all:

1. What are some of the key economic development goals of the country?
   1. What do you think is driving or motivating these goals?
   2. What do you think are the most useful government policies to achieve these goals?
2. What are some of the key social development goals (e.g. in health, education, or environmental protection as outlined in the Sustainable Development Goals) now being pursued by Zimbabwe?
   1. What is driving or motivating these goals?
   2. How would you describe the relationship between economic and social development (sustainable development) goals? (tensions, possible conflicts)
      1. How is the government managing the relationship between economic and social/sustainable development goals or priorities?
      2. Are there any institutional mechanisms that coordinate or bring together different sectors of government?
3. Where does agricultural policy fit within these differing development goals?
   1. What is your perspective on the future of agribusiness in Zimbabwe?
   2. What crops are the most promising for sustained agricultural development?
   3. How are farmers interests represented in Zimbabwe? (UNAC – farmers union)
      1. How are farmers organized?
   4. What would you identify as recent success stories in the farming sector?
      1. e.g. Which crops have been particularly successful? Why?
      2. What markers do you look for to determine success?
   5. How are the SDGs influencing government policies towards tobacco farming/production or promotion of alternative crops in Zimbabwe? (conserve water, preserve land, reduce hazardous chemical release, increase productivity and income for food producers)
   6. What role are the SDGs playing in tobacco control measures in the country?

One of the targets for the health SDG 3 is:

**3.a** Strengthen the implementation of the WHO Framework Convention on Tobacco Control in all countries, as appropriate.

- 1. How can the government reconcile population health goals with improving livelihoods of tobacco farmers?

1. What role does tobacco production play within these economic and social (sustainable) development goals? (Probe: Inquire about information sources and evidence base)
   1. What are our thoughts about tobacco farming in Zimbabwe at the present time?
   2. What contribution does tobacco farming make to Zimbabwe’s economic development goals (positive, negative, strong, weak…)?
   3. What support does Zimbabwe give to support tobacco growing as an economic development strategy?
   4. What incentives does the government provide to companies to establish tobacco processing and manufacturing (value-added)? (Probe: Ask to identify the policies to this end)
   5. Should Zimbabwe be providing any incentives to tobacco growing or manufacturing?
   6. How does the tobacco industry engage with government around agricultural, trade, tobacco farming or other related policies?
   7. What is the relationship between the government and the tobacco extension worker program? (This may not be relevant)
      1. How do government support for agriculture extend towards tobacco (e.g. input based?)
2. What role do tobacco control measures play within these economic development goals?
   1. What types of tobacco control measures are being pursued by the government?
   2. Where do you think the country is moving with tobacco control? Have you heard of the Framework Convention on Tobacco Control? What do you know about this treaty?
   3. What are some of the most promising tobacco control measures that could be considered by Zimbabwe (e.g. smoking restrictions, marketing restrictions, sponsorship restrictions, taxation [excise] policies, incentivizing alternative livelihoods)?
   4. Which of these measures are most likely to get widespread support and implementation?
   5. What is the status of tobacco consumption within Zimbabwe? (increasing, decreasing, about the same as for the past several years)
3. How would you describe the longer-term prospect of tobacco farming in Zimbabwe?
   1. What policies would you say would improve livelihoods of tobacco farmers?
   2. What policies is Zimbabwe currently pursuing to improve both the health and the livelihoods of tobacco farmers?
   3. How important do you think incentivizing alternative crops to tobacco would be to improve both the health and the livelihoods of tobacco farmers?
4. Relationship between private sector and government?
   1. Can you tell me about the private sector entities that you interact with in your work?
   2. What types of issues do you typically discuss?
   3. Are there formal mechanisms in place to facilitate these interactions?

Additional questions/probes by interview group:

**For *health* informants additional questions:**

1. What is the up to date status of tobacco control measures in Zimbabwe
2. How effective do you find engagement with other ministries whose primary concern is with economic development (or agricultural growth, trade, tobacco production/export (e.g. focus on the inter-ministerial liaison committee)?
3. How do they view tobacco control measures alongside the economics of tobacco farming?
4. What role are the new SDGs playing in advancing tobacco control measures in the country, or in improving engagement with other government sectors?
5. Can you describe your relationship with non-governmental organizations? Can you tell us of a time when this relationship helped move tobacco control forward?

For *tobacco farmer/industry* informants

1. How is the tobacco leaf industry doing in Zimbabwe at the moment (growing, declining, about the same as in recent years)?
2. How do they engage with governments in promoting the livelihoods of tobacco farmers?
3. How do they work with the tobacco industry or leaf-buyers to protect the livelihoods of tobacco farmers?
4. What activities do they undertake with tobacco farmers themselves to support their livelihoods?
5. How important is tobacco growing to the livelihoods of tobacco farmers?
6. How does tobacco compare with other crops or economic activities for the livelihoods of farmers?
7. How are you involved in identifying alternative crops to tobacco?
8. What concerns do you have in how tobacco control measures adopted by Zimbabwe might affect the livelihoods of tobacco farmers?

For *COMESA, SADC, WTO (+ bilaterals, mainly with countries in the region)*

1. How important is tobacco leaf production (tobacco manufacturing) in Zimbabwe’s regional and international trade?
2. How are tobacco control measures viewed within regional and international trade policy or agreements?
3. What positions on tobacco control measures have been taken (in COMESA, in SADC, by Zimbabwe at the WTO)? Can you tell us of a notable experience in one of these meetings where tobacco was discussed? What was happening? What was said?
4. How are tensions between tobacco control measures and tobacco trade being managed?
5. How do you see the economic agreements in which Zimbabwe participates (in COMESA, SADC, or WTO) improving the livelihoods of tobacco farmers?
6. How do you see the economic agreements in which Zimbabwe participates (in COMESA, SADC, or WTO) or in incentivizing a shift to alternative crops?
7. Do you think that these agreements have any impact on tobacco production and tobacco control? If so, how?
